# SNP rs10248565 in *HDAC9* as a novel genomic aberration biomarker of lung adenocarcinoma in non-smoking women

**DOI:** 10.1186/1423-0127-21-24

**Published:** 2014-03-21

**Authors:** Liang-Chuan Lai, Mong-Hsun Tsai, Pei-Chun Chen, Lee H Chen, Jen-Hao Hsiao, Shin-Kuang Chen, Tzu-Pin Lu, Jang-Ming Lee, Chung-Ping Hsu, Chuhsing K Hsiao, Eric Y Chuang

**Affiliations:** 1 Graduate Institute of Physiology, National Taiwan University, Taipei, Taiwan; 2 Bioinformatics and Biostatistics Core, Center of Genomic Medicine, National Taiwan University, Taipei, Taiwan; 3 Institute of Biotechnology, National Taiwan University, Taipei, Taiwan; 4 YongLin Biomedical Engineering Center, National Taiwan University, Taipei, Taiwan; 5 Graduate Institute of Biomedical Electronics and Bioinformatics, Department of Electrical Engineering, National Taiwan University, Taipei, Taiwan; 6 Department of Public Health, National Taiwan University, Taipei, Taiwan; 7 Department of Statistics and Informatics Science, Providence University, Taichung, Taiwan; 8 Department of Surgery, National Taiwan University Hospital, Taipei, Taiwan; 9 Division of Thoracic Surgery, Taichung Veterans General Hospital, Taichung, Taiwan

**Keywords:** Lung cancer, Microarray, rs10248565, *HDAC9*, Adenocarcinoma, Non-smoking

## Abstract

**Background:**

Numerous efforts have been made to elucidate the etiology and improve the treatment of lung cancer, but the overall five-year survival rate is still only 15%. Although cigarette smoking is the primary risk factor for lung cancer, only 7% of female lung cancer patients in Taiwan have a history of smoking. Since cancer results from progressive accumulation of genetic aberrations, genomic rearrangements may be early events in carcinogenesis.

**Results:**

In order to identify biomarkers of early-stage adenocarcinoma, the genome-wide DNA aberrations of 60 pairs of lung adenocarcinoma and adjacent normal lung tissue in non-smoking women were examined using Affymetrix Genome-Wide Human SNP 6.0 arrays. Common copy number variation (CNV) regions were identified by ≥30% of patients with copy number beyond 2 ± 0.5 of copy numbers for each single nucleotide polymorphism (SNP) and at least 100 continuous SNP variant loci. SNPs associated with lung adenocarcinoma were identified by McNemar’s test. Loss of heterozygosity (LOH) SNPs were identified in ≥18% of patients with LOH in the locus. Aberration of SNP rs10248565 at *HDAC9* in chromosome 7p21.1 was identified from concurrent analyses of CNVs, SNPs, and LOH.

**Conclusion:**

The results elucidate the genetic etiology of lung adenocarcinoma by demonstrating that SNP rs10248565 may be a potential biomarker of cancer susceptibility.

## Background

One of most commonly diagnosed cancers is lung cancer, which accounts for nearly 18% of all cancer-related deaths worldwide [1]. In the United States and other Western countries, the 5-year survival rate of lung cancer is only 15% and has not improved over several decades. In Taiwan, lung cancer mortality rates have become the highest in the world [2,3]. Even though numerous research efforts have been devoted to the development of lung cancer treatment over the past few decades, the overall 5-year survival rate is still as low as 15% [4].

Smoking is the primary risk factor for lung cancer [5]. In Western countries, 70-90% of lung cancers are attributable to cigarette smoking, whereas in Taiwan, only 7% of female lung cancer cases are associated with smoking [6,7]. Most non-smoking female patients with lung cancer have adenocarcinoma. However, the molecular mechanisms of lung adenocarcinoma in non-smoking women remain unclear.

Cancer appears to result from the progressive accumulation of genetic aberrations ranging from large, visible chromosome events to single nucleotide polymorphisms (SNPs). Genomic rearrangements that affect DNA sequences are called structural variants and include such things as insertions, deletions, duplications and inversions [8]. When the length of a structural variant is 1 kb or longer, it is defined as a copy number variation (CNV). CNVs have played important roles in recent cancer studies. Duplicated chromosomal regions may contain dominant oncogenes (e.g., *MYC* and *ERBB2*[9-11]), whereas deleted regions may harbor tumor suppressor genes (e.g., *RB1*, *CDKN2A*, and *PTEN*[9-15]). These genes play critical roles in multiple processes including cell growth, proliferation, apoptosis, and metastasis. For example, genomic imbalances and losses at 16q were shown to be associated with more poorly differentiated subtypes of prostate cancer [16]. DNA CNVs explained about 12% of the gene expression variations in breast cancer [17]. Concordant changes between mRNA expression levels and CNVs were observed in several genes located in copy number variable regions in lung cancer [18,19]. Furthermore, gene CNVs have been shown to be useful in predicting patient survival outcomes in lung cancer [20,21]. For example, the amplification and overexpression of epidermal growth factor receptor (*EGFR*) and the under-expression of dual specificity phosphates 4 (*DUSP4*) served as effective prognostic biomarkers in lung cancer [19,22]. Therefore, it is important to characterize DNA copy number changes for both the basic understanding of cancer and its diagnosis.

In this study, the genomic aberrations of lung adenocarcinoma in non-smoking women were examined using genome-wide human SNP arrays. The primary advantage of SNP arrays for our purposes was that the probe intensity of both alleles at each SNP allows the detection of CNV breakpoints and the estimation of the associated number of copies. In addition, loss of heterozygosity (LOH), used for surveying segments of allelic losses, can be examined by analyzing the genotype of both normal and lung tissues [23,24].

Although SNP genotyping is often used for examining the associations between cancer and normal tissues, the main focus of this study was not to identify the association of SNPs with lung adenocarcinoma in non-smoking women. Instead, we took advantage of the ability of whole-genome SNP arrays to concurrently analyze CNVs, SNPs, and LOH in order to identify the novel focal loci of lung adenocarcinoma. All results indicated that the SNP rs10248565 in *HDAC9*, the gene encoding histone deacetylase, was related to lung carcinogenesis. In this study, we demonstrated that SNP rs10248565 in *HDAC9* may be a potential biomarker for identifying important genetic determinants of cancer susceptibility and elucidating the genetic etiology of lung cancer in non-smoking females.

## Methods

### Sample collection

The study protocol was approved by the institutional review boards of National Taiwan University Hospital and Taichung Veterans General Hospital. The written consent form was approved by ethics committees, and all participants agreed with their written consents to participate in this study. In total, 120 pairs of cancer and normal lung tissue specimens were collected from non-smoking females. The selection criteria of clinical specimens depended on the pathology report, physical examination and cigarette smoking history. Surgical lung tissue specimens were immediately snap-frozen in liquid N_2_ and stored at -80°C until being further processed for DNA extraction. Only those paired samples passing quality checks (n = 61 pairs) were processed for SNP arrays.

### Isolation of genomic DNA, DNA amplification, labeling and hybridization of SNP arrays

Genomic DNA was isolated by phenol/chloroform extraction following standard protocols with 0.5% SDS and 200 μg/ml proteinase K. Total genomic DNA (250 ng) was digested with a restriction enzyme (*Nsp*I or *Sty*I) and ligated to adaptors that recognize the cohesive four bp overhangs. All fragments resulting from restriction enzyme digestion were substrates for adaptor ligation. A generic primer that recognizes the adaptor sequence was used to amplify adaptor-ligated DNA fragments. PCR conditions had been optimized to preferentially amplify fragments in the 200 to 1,100 bp size range. The amplified DNA was then fragmented, labeled, and hybridized to Genome-Wide Human SNP 6.0 arrays (Affymetrix, Inc., Santa Clara, CA). After 16 hours of hybridization at 49°C, the arrays were washed by Fluidics Station 450 and scanned by GeneChip Scanner 3000. Microarray data of this study are MIAME compliant, and have been submitted to the MIAME compliant Gene Expression Omnibus (GEO) database (accession number GSE33355).

### Copy number variation analysis

After scanning, the intensity data were analyzed by Partek® software (Partek®, St. Louis, MO, USA). Since both tumor and adjacent normal tissues were from the same individual, the reference baseline for each tumor tissue was its corresponding normal tissue. The criteria for searching for CNV regions in the whole genome were as follows: 1) copy number intensity ratio of tumor to normal tissue for each SNP was >2.5 or <1.5; 2) each individual had ≥100 continuous SNP variant loci; 3) the CNV regions existed in ≥30% of the study population. The overlapping genes located within the detected CNV regions were annotated using the documentation file version 30 provided by Affymetrix.

### TaqMan® copy number assays

TaqMan® assays were used to validate the total copy number of CNV regions. Total genomic DNA (20 ng; 5 ng/μl) was used for TaqMan® Copy Number assays (Life Technologies, Carlsbad, CA, USA). All reactions were performed in duplicate, including the FAM™ dye label-based assay for the target of interest and the VIC® dye label-based TaqMan® Copy Number Reference Assay. The TaqMan® probes for the target of interest were labeled with FAM at the 5′ end and linked by a non-fluorescent quencher at the 3′ end. RNase P labeled with VIC dye (Life Technologies) was utilized as the reference gene, which is known to exist in two copies in a diploid genome. All TaqMan® assays were performed following manufacturer’s instructions and copy number calculation was conducted by the delta-delta threshold cycle (∆∆Ct) method. PCR was performed with an Applied Biosystems 7900HT Fast Real-Time PCR System (Applied Biosystems, Carlsbad, CA, USA). Results were analyzed by CopyCaller™ version 1.0. Tumor samples with a delta Ct value between target and reference sequences were measured, and then compared to their paired normal samples.

### Single nucleotide polymorphism analysis

For SNP analysis, SNPs were obtained using Affymetrix® SNP Array 6.0 (each has more than 906,600 SNPs). After excluding SNPs with allele frequency <1% (157,703 SNPs) or call rate <90% (123 SNPs), 748,774 SNPs were further analyzed by McNemar-Bowker’s test to examine the difference of genotypes between normal and tumor tissues from the same subject. SNPs were coded according to the number of minor alleles, i.e., AA, Aa and aa, denoted as 0, 1, 2, respectively. The nonparametric McNemar-Bowker’s test was applied to examine the association between SNPs and tissues. The analyses were done in R version 2.9.0.

### Loss of heterozygosity analysis

Loss of heterozygosity (LOH) was defined as heterozygosity in normal tissue and homozygosity in tumor tissue. The genotypes between tumor tissue and its normal counterpart from the same subject were compared using Genome-Wide Human SNP 6.0 arrays (Affymetrix, Inc., Santa Clara, CA). LOH SNPs were identified in ≥18% of patients with LOH in the locus.

## Results

### DNA genetic aberration analysis

In this study, pairs of adenocarcinoma and adjacent normal lung tissue specimens were collected from 61 non-smoking women for the purpose of examining genome-wide DNA aberrations. The majority (72%; n = 44) of women were in early stages (I + II) and the mean (SD) age was 59.4 (11). Their clinical characteristics are listed in Table 1. CNV, SNP, and LOH were concurrently analyzed using Affymetrix Genome-Wide Human SNP 6.0 arrays. All chips’ call rates were greater than 99%.

**Table 1:** Characteristics of 61 non-smoking female lung adenocarcinoma patients

**Characteristics**	**Sample size**	**Age**
Female	61	59.4 ± 11
Tumor types		
Adenocarcinoma	61	59.4 ± 11
Tumor stage		
I + II	44	60 ± 11
III + IV	17	58 ± 11

### Copy number variation analysis

**Figure 1:**
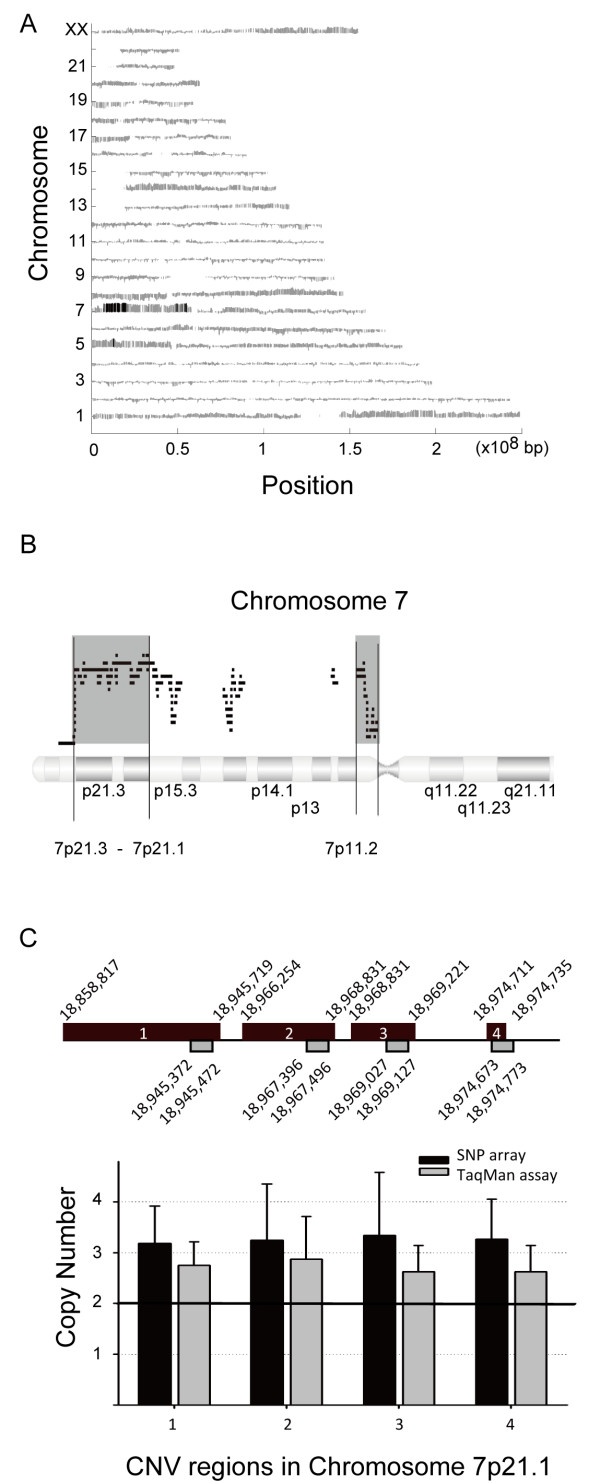
**Copy number variation analysis in non-smoking female lung adenocarcinoma patients. (A)** Distribution of genome-wide CNV using Affymetrix GeneChip® SNP 6.0 analysis. The criteria for the CNV regions were that SNPs must have copy numbers >2.5 or <1.5 and there must be at least 100 continuous SNP variant loci. Grey bars indicate regions with gains (above) or losses (below) in copy number. Black bars indicate that ≥30% of the patients had a particular CNV. **(B)** Common gain regions (≥30% of patients) of CNVs (grey area) were identified in 7p21.3-7p21.1 and 7p11.2. Black lines of each row indicate regions with copy number amplifications at >100 continuous SNP loci for each patient. **(C)** TaqMan assay validation of CNVs in chromosome 7p21.1. Four CNV regions (black block 1–4) identified by SNP arrays were examined using TaqMan® copy number assays (grey block).

We first identified common CNV regions among these lung adenocarcinoma samples. The criteria for searching the CNV regions in whole genome were stated in the Methods section. In total, there were 424 CNV regions. Figure 1A shows the distribution of CNV for each chromosome among 61 paired samples. Each grey bar indicates the amplification or deletion regions in tumor tissue. Black bars indicate where >30% of patients (n > 18) had CNV. An expanded view of these results showed that one third or more of these patients had a genetic amplification at 7p21.3-7p21.1 and 7p11.2 (Figure 1B). In contrast, no common deletion regions were identified.

In order to validate the common amplification regions, four CNV regions in 7p21.1 were chosen for TaqMan® copy number assays. In the upper panel of Figure 1C, the positions of 4 CNV regions (black blocks) identified by SNP arrays and those examined by TaqMan® copy number assays (grey blocks) are shown. The TaqMan assays showed that the copy numbers in all 4 regions were greater than normal (lower panel of Figure 1C), indicating these regions are common amplification regions in non-smoking female lung cancer patients.

In order to understand the function of genes in common amplification regions, functional analysis was done using Ingenuity Pathway Analysis (IPA). The results revealed that the common amplification regions contain 29 genes. Network analysis showed that these genes were mainly involved in cellular development, cellular growth and proliferation, and cancer. Among these 29 genes, *EGFR* (encoding epidermal growth factor receptor) and *HDAC9* were previously reported to have an association with lung tumorigenesis.

### Single nucleotide polymorphism analysis

Next, genotyping of SNPs was analyzed in normal and adenocarcinoma tissues from the same subject. SNPs with minor allele frequency <0.01 were excluded. After excluding SNPs with low minor allele frequency (181,503 SNPs) or SNPs with departing Hardy-Weinberg Equilibrium (*P*-value <0.0001; 2,816 SNPs), the remaining 684,877 SNPs on autosomal chromosomes were further analyzed by McNemar-Bowker’s test to examine the differences in genotypes.

**Figure 2:**
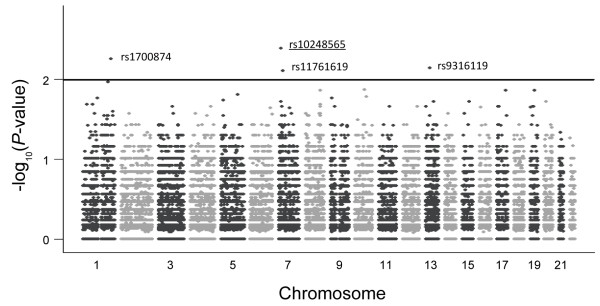
**Single nucleotide polymorphism analysis in non-smoking female lung adenocarcinoma patients.** Each dot denotes a SNP. The distribution of –log(*P*-value) of each SNP was plotted across chromosomes. The four SNPs with *P*-values smaller than 10^-2^ are labeled.

Since this study adopted a paired design, which provides less variation than general case–control studies and can achieve a higher statistical power, a strict criterion of *P*-values, Bonferroni correction, was not performed. As shown in Figure 2, a Manhattan plot showed that there were four SNPs with *P*-values smaller than 0.01. Each dot represents a SNP. The distribution of –log(*P*-value) of each SNP was plotted across chromosomes. Information on these four SNPs (rs1700874, rs10248565, rs11761619, and rs9316119) is listed in Table 2. Only rs1700874 was located in an intergenic region (between *TGFB2* and *LYPLAL1*); the rest of the SNPs were located in introns. Among them, the SNP with the lowest *P*-value, rs10248565, is located in an intron of *HDAC9*.

**Table 2:** SNPs significantly associated with lung tumors in non-smoking female patients

**SNP**	**Chromosome**	**Position**	***P*-value**	**Location**
rs1700874	1	219,182,858	4 × 10^-3^	Intergenic *TGFB2 & LYPLAL1*
rs10248565	7	18,974,723	3 × 10^-3^	*HDAC9* intron
rs11761619	7	33,549,392	8 × 10^-3^	*BBS9* intron
rs9316119	13	45,696,862	6 × 10^-3^	*GTF2F2* intron

### Loss of heterozygosity analysis

**Figure 3:**
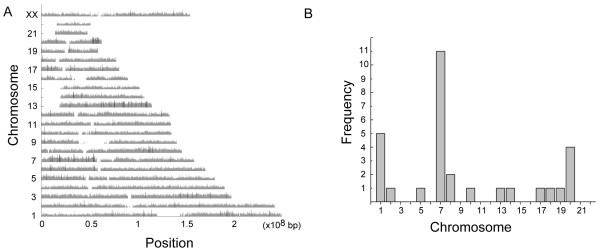
**Loss of heterozygosity analysis in non-smoking female lung adenocarcinoma patients. (A)** Distribution of LOH using Affymetrix GeneChip® SNP 6.0 analysis. Grey bars indicate regions with LOH SNPs. Black bars indicate that >18% of total patients (n > 11) had a particular LOH SNP. **(B)** Frequency of LOH SNPs in each chromosome.

Lastly, we examined the distribution of LOH in each chromosome (Figure 3). Because the proportion of LOH loci ranged from 10% to over 50%, LOH for a SNP was defined as at least 18% of patients (≥11 patients) with LOH in the locus. As shown in Figure 3A, black bars indicate ≥18% of patients with the LOH SNP. In total, there were 30 SNPs indicating LOH. Most of these SNPs appeared in chromosome 7 (Figure 3B).

**Figure 4:**
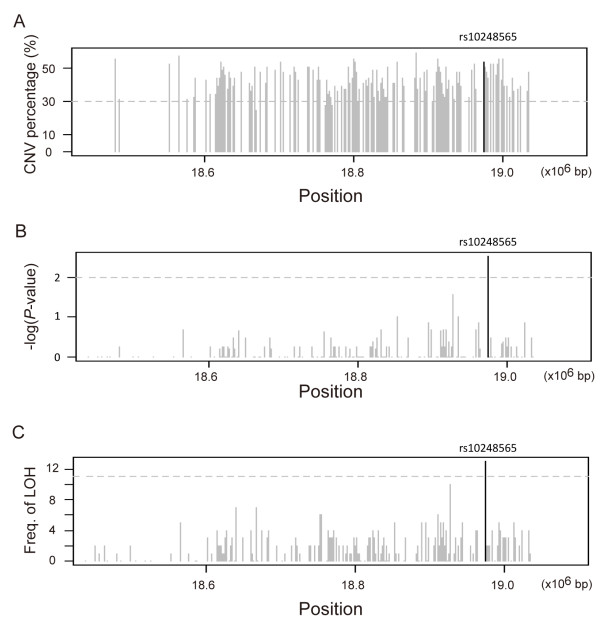
**Genomic aberration of SNP rs10248565 was identified using (A) CNV, (B) SNP, and (C) LOH analyses.** In each panel, SNP rs10248565 is indicated by a black bar.

Among these LOH SNPs, we noticed that SNP rs10248565 was associated with lung adenocarcinoma and was located in the CNV region. Combining the results of CNV, SNP, and LOH analyses (Figure 4), we concluded that rs10248565 is a possible biomarker of lung adenocarcinoma in non-smoking females.

## Discussion

It is well-known that there are many causative elements in cancer progression and tumorigenesis, such as sequence mutations, transcriptional alterations, and genomic changes. Among these complicated factors, structural variations of DNA sequences have been widely reported to serve as a key driver to dysregulate the transcriptome during tumorigenesis [17]. Furthermore, since genes located within the variable regions are candidate oncogenes or tumor suppressors, an integrative analysis of CNV, SNP, and LOH may provide more information in dissecting the lung tumorigenic process. To help explain the relationship between copy number and gene expression, we performed an integrative analysis in paired lung adenocarcinoma tissue specimens to identify genomic alterations in tumor tissues.

Our CNV results showed that at least 30% of the samples had amplifications at chromosomes 7p21.3-7p21.1 and 7p11.2. However, no deletion regions were identified. This may due to the stringent selection criteria adopted here. Comparing with other studies, several aberrant regions have been detected using high resolution karyotyping techniques to scan lung cancer genome, such as amplifications of 3p25-27 and 5p13-14, and deletions of 3p21 and 9q21 [25]. Several studies reported that a deletion on chromosome 5q in small cell and squamous cell lung cancer subtypes may be associated with smoking history [26-29]. Conversely, amplifications of 5q have been detected in adenocarcinoma [30,31]. We did not observe any amplification regions in chromosome 5 in this study, which may be explained by differences in experimental design, selection criteria, and ethnicity of study populations.

Further investigations of the 29 genes located within these CNV regions identified several key players involved in the tumorigenic process. For instance, loss of docking protein 2 (*DOK2*) as well as expression of baculoviral IAP repeat-containing 2/3 (*BIRC2*/*3*) can facilitate lung cancer cell proliferation and contribute to lung tumor development [32,33]. EGFR is involved in the signal transduction pathways of cell proliferation, differentiation, adhesion, protection from apoptosis and survival. Numerous reports have shown that EGFR gene mutations are frequently detected in lung cancer, especially in adenocarcinoma, females, and non-smoking patients [34].

The gene encoding HDAC in chromosome 7p21.1 was identified in all CNV, SNP and LOH analyses and is worthy of mention here. HDAC is involved in deacetylation of lysine residues in the N-terminal tails of nucleosomal core histones [35], and it has also been implicated in the development of cancer [36]. The activity of several tumor suppressors is regulated in part by HDACs, such as p53 binding protein that regulates cell cycling in response to DNA damage [37]. HDAC inhibitors were developed as anti-cancer agents with a high degree of selectivity for killing cancer cells. In one study, inhibition of HDAC induced DNA damage which only normal cells, but not cancer cells, can repair, and resulted in cancer cell death [38]. Inhibition of *HDAC6* significantly enhanced cell death induced by the topoisomerase II inhibitors in transformed cells, but not in normal cells [21]. Inhibition of *HDAC1* and *HDAC2* enhanced the radiosensitivity of non-small cell lung cancer [39]. Unfortunately, the expression levels of *HDAC9* did not differ significantly between tumor tissue and adjacent normal tissue in our study (data not shown). This may be due to the location of the SNP in an intron of *HDAC9*, and further investigation of the mechanism of genomic aberration in *HDAC9* is warranted.

The hypothesis underlying our SNP analysis was that if SNPs were associated with cancer, the proportions of different alleles would be different in cancer and normal groups. Previously, rs7086803 at 10q25.2, rs9387478 at 6q22.2 and rs2395185 at 6p21.32 were identified as lung cancer susceptibility loci in never-smoking women in Asia [40]. In this study, we identified another 4 SNPs (rs1700874, rs10248565, rs11761619, and rs9316119) that were significantly (*P* <0.01) associated with lung cancer. SNP rs1700874 is located at an intergenic region in 1q41 between *TGFB2* and *LYPLAL1*. The transforming growth factor beta family plays an important role in cell cycling, cell growth, apoptosis, and protein synthesis, and is therefore involved in many pathological processes [41,42]. A previous study showed that *TGFB2* may correlate with heart disease and pulmonary function in mice [43]. The function of *LYPLAL1* is still unclear. SNP rs10248565 is located in 7p21.1 within *HDAC9*, the significance of which was discussed above. SNP rs11761619 is in 7p14.3 within *BBS9. BBS9* is associated with kidney and ovarian diseases [44,45], and may be a tumor suppressor gene for Wilms’ tumor [46]. SNP rs9316119 is in 13q14.12 within *GTF2F2*, which is known to affect the progression and survival of epithelial ovarian cancer [47].

In this study, we identified 30 SNPs with LOH. Most of these LOH SNPs were located in chromosome 7 (Figure 3B). LOH analysis has been used to identify genomic aberrations in previous studies. For instance, loss of heterozygosity at chromosomal regions 3p21.3 (site of *RASSF1A*, a member of the Ras association domain family, and *FUS1*), 3p14.2 (*FHIT*, a fragile histidine triad gene), 9p21 (*p16*), and 17p13 (*p53*) was identified as an early event in the development of non-small cell lung cancer [48].

It may seem contradictory that the SNP rs10248565 in *HDAC9* was increased in copy number but also showed loss of heterozygosity. The observed LOH accompanied by a gain in copy number may result from preferential amplification of one parental allele, because CNV analysis cannot identify situations in which the loss of one allele is followed by duplication of the remaining allele. Also, LOH cannot detect any amplification that might be involved in pathogenesis. Therefore, we conducted a concurrent LOH and CNV analysis with the expectation of more precisely defining the nature of genomic alternations observed in either analysis alone.

## Conclusion

In conclusion, the high mortality of lung cancer worldwide is largely attributable to the difficulty of obtaining an early diagnosis and the lack of effective therapeutic methods. To improve survival rates in non-smoking lung cancer patients, a comprehensive analysis of the molecular signature of the carcinogenic processes in adenocarcinoma in non-smoking Taiwanese women was conducted to identify novel biomarkers for diagnosis and new molecular targets for drug development. Although more studies are still needed, SNP rs10248565 in *HDAC9* may be one of the potential biomarkers for lung adenocarcinoma in non-smoking women.

Microarray data from this study have been submitted to the Gene Expression Omnibus database (accession number GSE33355).

## Competing interests

The authors declare that they have no competing interests.

## Authors’ contributions

LCL, MHT, CKH, and EYC provided conception and design. LCL, CKH, and EYC provided financial support. JML and CPH provided study materials and patients. PCC and SKC collected and assembled data. LCL, MHT, PCC, LHC, JHH, TPL, and EYC analyzed and interpreted data. LCL, MHT, PCC, and EYC wrote manuscript. All authors read and approved the final manuscript.
